# Sterility testing of germ-free mouse colonies

**DOI:** 10.3389/fimmu.2023.1275109

**Published:** 2023-11-07

**Authors:** Olga Dremova, Maximilian Mimmler, Nadja Paeslack, My Phung Khuu, Zhenling Gao, Markus Bosmann, Lucien P. Garo, Nathalie Schön, Alexa Mechler, Yunes Beneich, Vivian Rebling, Amrit Mann, Giulia Pontarollo, Klytaimnistra Kiouptsi, Christoph Reinhardt

**Affiliations:** ^1^ Center for Thrombosis and Hemostasis (CTH), University Medical Center of the Johannes Gutenberg-University Mainz, Mainz, Germany; ^2^ Pulmonary Center, Department of Medicine, Boston University School of Medicine, Boston, MA, United States; ^3^ German Center for Cardiovascular Research (DZHK), University Medical Center of the Johannes Gutenberg-University Mainz, Partner Site Rhine-Main, Mainz, Germany

**Keywords:** germ-free, gnotobiology, microbiota, isolator, sterility, contaminant, culture

## Abstract

In biomedical research, germ-free and gnotobiotic mouse models enable the mechanistic investigation of microbiota-host interactions and their role on (patho)physiology. Throughout any gnotobiotic experiment, standardized and periodic microbiological testing of defined gnotobiotic housing conditions is a key requirement. Here, we review basic principles of germ-free isolator technology, the suitability of various sterilization methods, and the use of sterility testing methods to monitor germ-free mouse colonies. We also discuss their effectiveness and limitations, and share the experience with protocols used in our facility. In addition, possible sources of isolator contamination are discussed and an overview of reported contaminants is provided.

## 1 Introduction

The microbiome is a dynamic and interactive micro-ecosystem that is integrated in macro-ecosystems, including eukaryotic hosts (1). Notwithstanding the large quantities of omics-data generated over the past decade, the functional roles that individual microorganisms exert on the other members of this complex ecosystem, including the physiology of the host, remain poorly understood. The physiology of animal metaorganisms is strongly influenced by their microbiota (2). These influences range from local effects on the intestinal mucosa to systemic maturation of immune functions and inflammaging, or regulation and interference with host metabolic functions (3–7). Of note, microbiome composition is influenced by pharmacotherapy (e.g., antibiotics), and even more interesting, the efficacy of various pharmacological treatments might be influenced by microbiota composition (8, 9). Therefore, there currently is an unmet need to move from association-based evidence to causality, and to pinpoint the exact molecular mechanisms underlying microbiota-host interactions in health and disease. To complement results from sequencing studies, researchers must perform experimentation on well-defined gnotobiotic rodent models that have an annotated and controlled colonization status.

Germ-free (axenic) mouse models are crucial for gnotobiotic experimentation and the exploration of microbiota-host interactions (10, 11). The strength of these mammalian model organisms is the separation of the host from its colonizing microbiota, consisting predominantly of bacterial communities, and to a lesser extent of fungi, viruses, and protozoa (12, 13). Hence, germ-free mice enable the functional investigation of how commensals interfere with adaptive processes, cell-based mechanisms, and biochemical pathways (14). Gnotobiotic experimentation includes the association of germ-free mice with defined microorganisms, synthetic communities (syncoms), or complex microbiota (e.g., by fecal microbial transplantation) (15). This approach is essential to causally address how defined microorganisms or complex microbiomes impact various aspects of host physiology (16). Hence, gnotobiotic experimentation complements taxonomic sequencing technologies, yielding correlation-based evidence reported by a myriad of clinical and mouse microbiome studies (17, 18).

Enormous variation in this complex microbial ecosystem, which probably becomes most apparent when comparing the same laboratory mouse strain, with the same genetic background, kept at different husbandries and analyzed under well-standardized conditions, has been reported (19). Therefore, association studies based on taxonomic sequencing are insufficient to unravel how microbial communities interfere with host physiology. This is especially relevant for human microbiome studies since host genetics have been reported to have a minor role in determining microbiome composition, whereas environmental factors have been found decisive (20, 21). Interestingly, even genetic disease models depend on the colonization status of the host (22). Therefore, despite higher costs, gnotobiotic experimentation with germ-free mouse models associated with well-defined model microbiomes or individual microbial species constitutes a key technology needed to disentangle specific functional roles of the microbiota (23).

Gnotobiotics evolved in the late nineteenth century, shortly after the scientific debates on germ-free life, when the first germ-free rederivation experiments on guinea pigs were reported by George Nuttall and Hans Thierfelder in Berlin (24), followed by rederivation experiments on chicken and goats (25, 26). However, since germ-free isolator technology had yet to be developed, these first germ-free animals were prone to microbial contamination. In 1946, the group of James A. Reyniers, at the Laboratory of Bacteriology at University of Notre Dame (LOBUND) in Illinois (USA), succeeded in establishing gnotobiotic steel isolators combined with an autoclave to maintain successive generations of germ-free rodents (27). Rearing germ-free albino rats required cesarean-derivation and subsequent hand feeding (27, 28). To rear germ-free rats, a stainless-steel isolator system, that was autoclaved in a large steam autoclave, was likewise developed by Bengt Gutsafsson at the University of Lund in Sweden (29, 30). Next, Philip C. Trexler developed the flexible film isolator system, which worked without autoclaving, and instead was based on germicides and had a controlled airflow (31). Another technical improvement was the possibility of keeping gnotobiotic mice in microisolator cages, but with a higher risk for contamination (32, 33). Meanwhile, embryo transfer efficiently applied for rederiving first generation germ-free mouse lines and various germ-free inbred mouse strains have become commercially available and can be securely transported using germ-free shippers (34–36).

Germ-free mice are certainly key as a model system to improve our understanding of gut microbial ecology (37). Gnotobiotic isolator technology enables the intentional colonization of germ-free mice with a complex gut microbiota (e.g., cecal contents from a conventionally raised donor mouse), consecutive colonization with individual microbes (monocolonization) (38, 39), or a defined set of selected bacteria from pure cultures (40). There is growing interest in studying gnotobiotic mouse models colonized with minimal microbiomes, so-called synthetic microbiomes (synthetic communities; syncoms) (15, 41), in order to limit experimental variability and to improve the reproducibility of rodent studies. The altered Schaedler flora (ASF) is probably the most prominent example of a standardized model microbiome, consisting of eight culturable and quantifiable bacterial species (42–45). Meanwhile, additional syncoms have been developed (e.g., Oligo-Mouse Microbiota (OMM), Simplified Human Intestinal Microbiota (SIHUMI), and Simplified Intestinal Microbiota (SIM)) (46–49). Another application is the use of germ-free rodent models for fecal microbiota transplantation (FMT) studies, using microbiomes from human donors or inter-species microbiota transplantation models (50–52). Preclinical studies, with the aim to study the microbiota of human donors with certain physiologic or disease phenotypes, are based on the transplantation of human gut microbiota into germ-free mouse models to transmit donor traits (humanized gnotobiotic mouse models) (53–55). However, the genetic background of the recipient rodent system strongly influences the composition of the transferred microbiota in the gnotobiotic host (51). In all these aspects, germ-free mouse isolator technology is superior to microbiota depletion using various antibiotic regimens, particularly since antibiotics evoke additional effects on host physiology that are independent of the host colonization status (23, 56).

Naturally, germ-free mice are sterile only within the limitations of the sterility testing methods applied. Unfortunately, these methods are still not standardized between different gnotobiotic facilities. In this regard, James A. Reyniers noted in his conference report in 1959 that *“the science or art of detecting contamination is always the limiting factor and is at best a temporary situation”* (57). It should be appreciated that germ-free housing conditions are greatly influenced by variations in the diet itself (i.e., batch-to-batch variation), irradiation procedures on the breeding diet (e.g., gamma *vs*. electron beam radiation, radiation dose), and the autoclaving protocols applied (e.g., the autoclave, autoclaving program, temperatures, and steam pressure). In addition to the training and experience of personnel and the routines for operating germ-free isolator technology, these parameters may vary between different facilities. Although routine protocols to control the microbiological status have been established in different germ-free facilities (58), they have not been well-described in much detail and still vary. We here provide a literature-based review, describing what is known on gnotobiotic isolator technology, the efficacy of sterilization methods, possible sources of contamination, and applicable sterility testing procedures.

## 2 Features of germ-free mouse isolator technology

### 2.1 Germ-free rederivation

The first germ-free animals were derived by hysterectomy and were hand-raised, which was successfully performed by the Laboratory of Bacteriology at the University of Notre Dame (28, 59, 60). Nowadays, hysterectomy and embryo transfer are the most used methods to generate germ-free mice from conventionally raised (CONV-R) stock (11, 34, 35). Rederivation by hysterectomy begins with the synchronous and timed mating of CONV-R donor and germ-free recipient breeding pairs. Shortly before birth, the uterus containing the pups, which is sterile, is clamped off and removed from the CONV-R donor’s abdomen. The uterus is then transferred into a special rederivation isolator via a dip tank containing disinfectant (e.g., iodine or 10% bleach) (11). In the destination isolator, the germ-free foster mother (recipient), which recently gave birth itself, fosters the freshly delivered pups (11, 60). After weaning, the freshly rederived mice can be mated inside a sterile isolator to start a new germ-free colony of the strain or genotype of interest. Alternatively, rederivation can be executed by embryo transfer. Here, embryos in the two-cell state are implanted into the oviduct of a germ-free surrogate mother, which will later give birth inside a sterile isolator (34, 35). A more detailed description of these methods can be found elsewhere (60, 61). As rederivation methods are highly delicate procedures that require trained staff and special equipment, most germ-free facilities choose not to perform these procedures themselves. Instead, companies have commercialized the rederivation of germ-free animals for users. These generated germ-free animals are transported in specially designed germ-free shippers to the destination facility, ensuring the germ-free state of the animals (36).

### 2.2 General principles of isolator technology

**Figure 1:**
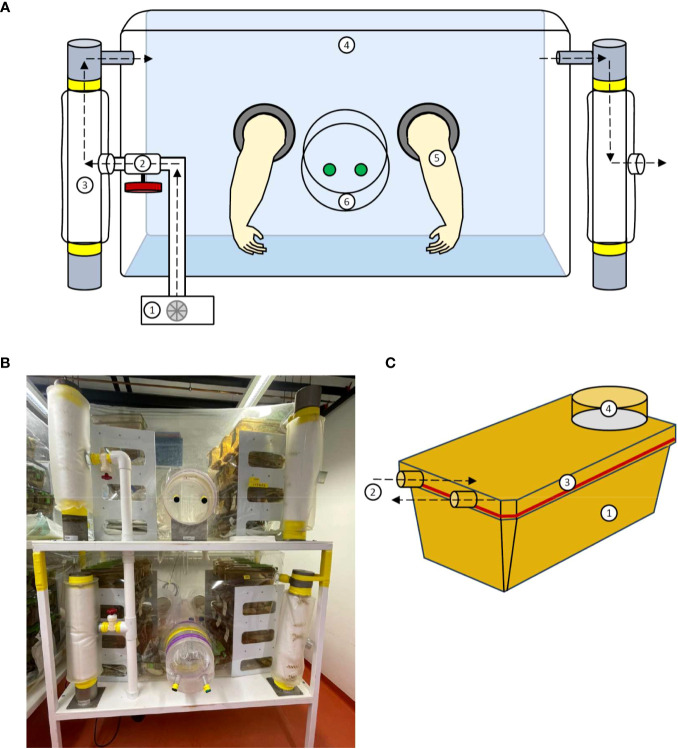
Structure of a flexible film isolator and the ISOcage P. **(A)** Schematic view of the isolator construction including the 1. Blower; 2. Air valve; 3. Filter column; 4. Isolator shell; 5. Gloves and 6. Isolator port closed with caps from in- and outside. **(B)** Photograph of a flexible film isolator. **(C)** Schematic view of the ISOcage P system with 1. Cage, 2. Air in- and outlet connecting to cage rack, 3. Hermetically sealed lid and 4. HEPA air filter (Tecniplast ISOcage P system). Air flow is depicted by dotted arrows.

To maintain a rederived germ-free mouse colony, sterile long-term housing is necessary. Here, an isolator creates an impermeable mechanical barrier separating its sterile inner environment from the outside. Transparent flexible-film isolators made of polyvinyl chloride (PVC) with positive pressure are widely used to provide a sterile or microbially controlled environment, which ensure enhanced visibility and more operational space (62–64). Isolator components have remained consistent throughout the different generations of isolators, including essential components such as the isolation chamber, air filter system, port system, blower, and gloves, which are illustrated in **Figures 1A, B**. The large housing unit of the isolator provides enough space for mouse cages and all supplies needed for animal care and experimental procedures. Two to three-tiered shelves in the isolation chamber increase the vertical storage space for animal cages. The isolator floor is covered by a robust extra canvas, protecting the isolator shell, and facilitating the work in the isolator. The isolator chamber is connected to an air-filter system. The blower inlet aspirates air into a column wrapped in filter cloth. A ball valve allows adjustment of air pressure going into the isolator, which facilitates the work in a semi-inflated isolator (i.e., by facilitating access to distant points). Filtered air, free of microorganisms and spores, then enters the isolator cavity, providing sterile air to the residing animals and inflating the isolator shell, which keeps the inner pressure positive. The positive pressure principle prevents the entry of microorganisms through microscopic leaks in the shell (11). Outgoing air is passively dissipated via a separate filter column, thus shielding the sterile interior from entering environmental microorganisms. Gloves made of latex, polyurethane, or nitrile rubber are mounted and hermetically sealed at the side of the isolator (62). This enables the operator to handle gnotobiotic animals in the isolator while shielding them from exposure to the environment. A rigid transfer port within reach of the gloves is installed in the isolator shell. This connects the sterile inner environment to the outside and is sealed with plastic caps from both sides (one cap on the inside of isolator and one on the outside, both secured by rubber bands). The double-doored port enables the isolator to be opened stepwise while keeping contaminations outside the sterile chamber. The isolator can thus be loaded by connecting autoclave sterilizing cylinders with sterile goods to the transfer port by a transfer sleeve with nipples. The created channel is then filled with microbicidal vapors/fog and incubated, thus creating a sterile lock, through which autoclaved material can be transferred from the cylinder into the channel and imported into the isolator chamber. This allows animal care supplies and experimental materials to be imported into the isolator. The isolator is usually placed on a table fitted with bidirectional locking casters that secure the structure during work and prevent it from moving.

### 2.3 The ISOcage P system

As an alternative to isolators, the ISOcage P system was developed to provide sterile isolation at the cage level (**Figure 1C**) (65). Air flow is mechanically driven by the ventilation system of the cage storing rack, whereby air passes through a high-efficiency particulate air (HEPA) filter into the sterile individually ventilated cage (IVC). The air inlet and outlet of the ISOcage P are equipped with double gasket self-closing nozzles, which close automatically when the cage is removed from the ventilation rack. Thus, the hermetic cage remains pressurized, which prevents air exchange between the cage and the environment, mimicking the positive pressure isolator principle. The individual ventilation of the ISOcage P protects different mouse cohorts from one another by preventing cage-to-cage contamination. This is a known problem in the isolator system. In the event of contamination, all open housing cages within the isolator chamber are affected. Depending on the experimental design, separate sterile cage animal housing can be of great advantage. As the cage isolators comprise a single sterile unit, it allows multiple experimental conditions to be conducted simultaneously in the same rack achieving an IVC-like density. Furthermore, experiments of mono-association of axenic mice with a single microbe or multiple defined microbiotas can be performed in a cage-restricted manner. After completion of an experiment,contaminated cages can easily be sterilized and are ready to be used again. In contrast, monocolonized isolator units require complete reconstruction and sterilization, which is an expensive and time-consuming procedure. However, because sterile housing cages need to be changed or opened for experimental operations more frequently, they are susceptible to contamination. Therefore, handling and opening sterile cages needs to be strictly standardized and performed under a sterile class II biosafety cabinet, with operators wearing adequate protection clothing (11).

Overall, the ISOcage P system provides reliable bioexclusion, nearly as good as the isolator concept (32). It is highly suitable for housing gnotobiotic and immunocompromised animals by shielding them from environmental influences. It boasts advantages in ergonomics, flexibility, and density, while also increasing the number of simultaneously feasible studies with different conditions. However, for long-term housing and breeding germ-free animals, the ISOcage P system is not the optimal choice, as the handling and opening of the ISOcage P constitutes a comparatively high risk for contamination, which is safer to be performed in a sterile isolator. Therefore, it might be worth considering combining both systems within one’s gnotobiotic facility, thus keeping the breeding and stock of germ-free animals in sterile isolators and transferring them into the ISOcage P system when performing experiments.

The ISOcage P system, which provides sterile isolation at the cage level, comes with different advantages and disadvantages. Because different mouse cohorts are separated from each other on cage level, accompanied by individual cage ventilation, cage-to-cage contamination can be prevented. This is especially useful for short term experiments, as single sterile cage units allow the simultaneous conduction of experiments, including multiple conditions in the same rack achieving IVC-like density. Importantly, the necessity of frequent cage changing makes them susceptible to contamination. Thus, their use is especially suited for short-time periods to keep the opening of cages and potential contamination at a minimal level, while exploiting the advantage of the easy handling. On the contrary, the long-term husbandry and breeding of mice requires frequent opening and changing of cages, which is therefore more suitable to be performed in the sterile environment of a flexible film isolator, minimizing the risk of contamination. Here, the precise handling, which is needed for experimental operations, is negligible.

### 2.4 Staff requirements

In order to house and breed germ-free animals in sterile isolators, trained animal caretakers are required to perform daily basic animal care including bedding changes, feeding, breeding, weaning, and organizing stock. Depending on the number of animals housed in the facility, several animal caretakers may be needed. Additionally, experimenters and assistants are involved in planning and performing experiments in the isolators (e.g., mono-associations, feeding experiments, or behavior experiments). Here, trained staff must assess welfare of experimental animals on a daily basis (e.g., by scoring and weighing). Overall, special training is required for all staff employed in a germ-free facility maintaining isolators regarding sterilizing and autoclaving procedures, importing and exporting materials and animals into/from isolators, connecting isolators, sterility testing, and assembling/disassembling the isolator constructions. Furthermore, employment of a veterinarian can be of great advantage for optimizing animal welfare.

## 3 Applicable sterilization methods

There are several methods applied to maintain the sterile barrier. However, not all of them apply to every material required for maintenance of a sterile environment. A key aspect of gnotobiotic technology is the sterilization of material, including not only the film isolators, diet, water, and bedding, but also the associated equipment required for experimental procedures such as needles or scales.

The first step to establish a sterile environment is the sterilization of the film isolators, which will host the germ-free animals. This process is usually performed using chemical antimicrobial compounds applied with a spray gun to corroborate the spreading of disinfectant throughout isolator surfaces. The first attempts to maintain a sterile environment utilized peracetic acid (63, 64). However, the highly corrosive nature of this chemical raised the need to apply less aggressive disinfectants such as alcide (66, 67). Other approaches included sterilization by ethylene oxide gas (68) or formaldehyde gas (69). Nowadays, in order to avoid toxicity and to achieve more safety and cost efficiency, chemical compounds such as chlorine-oxide products are commonly used, which have proved to be efficient against both bacteria and spores (70). Another benefit of chlorine-oxide products is their fogging capacity (70).

Maintaining a sterile isolated environment to host animals is not the only challenge of gnotobiotic facilities. Long-term housing of gnotobiotic animals requires the maintenance of axenic conditions. This cannot be achieved without a large autoclave with sufficient capacity to fit the transfer cylinders, large metal autoclavable cylinders that are used to supply isolators with required sterilized material. The most popular way to insert material in an isolator is to pack it in the cylinders, which are safely sealed until connected to the isolators, although there are facilities that have developed protocols avoiding the use of such cylinders (71). Autoclaving is accomplished by steam sterilization, a process that combines temperature, steam, pressure, and time to eliminate microbial life. This is suitable for autoclavable material like cages, bedding, water, metal instrumentation, and food. The use of biological and chemical indicators is highly recommended, although they often do not meet standards (72, 73). A challenging issue regarding the housing of gnotobiotic animals is the supply of food, which is often a source of contamination (74). Diet consistency is an important factor for diet selection. While the most common form used for laboratory animals is a pelleted diet because it is easy to handle and store and has reduced dust in the facilities, (75) extruded diets are preferable for gnotobiotic facilities. They are baked at high temperatures during the manufacturing process, thus reducing in advance the bacterial load of the natural products used in the feed manufacturing. Moreover, extruded diets are less dense (75) and since steam sterilization process depends on proper steam penetration in the food, the sterilization process of extruded diet is more effective. However, the baking process of the extruded diet is not sufficient to eliminate the bacterial load of the diet’s ingredients. The food used for the gnotobiotic animals needs to be further sterilized by autoclaving and/or γ-irradiation (> 25 kGy) to achieve full sterility. However, γ-irradiation is only recommended for extruded diets because the bacterial load in pelleted diets is too high to be eliminated by irradiation (75). Irradiated diet is easier to handle and occasionally preferred, especially when autoclaving is not an option (76, 77). By irradiation, the nutrients of the diet are not affected the way they are by autoclaving. Furthermore, the irradiation companies test irradiated material for bacterial contaminants and provide certificates for the sterility of their food. However, risk of contamination remains because of the presence of radioresistant bacteria (e.g., *Deinococcus radiodurans* can survive 17.5 kGy) (78). Additionally, irradiation may have adverse effects on research outcomes. For instance, a study by Prasain et al. in 2017 demonstrated that irradiation could lead to an increase in oxidized lipid metabolites (79). On the other hand, autoclaving food is laborsome, expensive, and often needs to be validated, both by validating the recorded temperature and pressure of the autoclave cycles and the steam autoclave itself, and also the samples by using biological or chemical indicators to prove sterilization efficacy (58). After autoclaving, the diet is hardened, difficult to gnaw, and the concentration of heat-sensitive diet ingredients must be adjusted to compensate for the loss of some nutrients through the sterilization process, in a way that the nutrient requirements of the animals are met (80).

## 4 Relevant sources of isolator contaminations

Maintaining germ-free animals requires an isolator system that effectively segregates sterile animals from ubiquitous microorganisms present in the external environment. Since preserving a germ-free husbandry entails a multitude of materials, technical knowledge, and well-trained staff, the sources of isolator contaminations are manifold. Failure of one parameter is sufficient to contaminate an entire isolator, including its animals. Moreover, the rebuilding of the isolators and eventual (re)generation of germ-free mice through derivation is a costly and time-consuming process.

Contamination of a germ-free system can occur through damage of the physical barrier or by introducing improperly sterilized material. The former includes leaks in the plastic film isolator, gaskets, port caps, or rubber gloves. Prolonged breaks of positive pressure in isolators harbors the risk of aerosol translocation of contaminants through small holes in the material. Gloves especially should be considered a weak point. As they are frequently worn and stretched out due to frequent use and animal contact, gloves need to be inspected for leaks or signs of material fatigue on a regular basis. Chlorosulfonated polyethylene (CSM) is a widely used material for gloves due to its chemical resistance and high tensile strength. Nonetheless, frequent inspection and replacement can prevent transfer of contaminants, such as commensal skin bacteria (58).

Besides damage of the isolation system, contamination can occur due to improper sterilization. Inadequate monitoring of each sterilization cycle performance, and technical failures in sterilization apparatuses, can lead to the persistence of microorganisms or their spores. For instance, the spore former, *Clostridium perfringens type D*, was found in autoclaved mouse pellets after an ineffective autoclaving process. Wet steam containing 5% entrained water resulted in lower heat transfer efficiency (81). Sterilization is usually performed by autoclaving, irradiation, or spraying with decontaminating gases. Autoclaving is performed to sterilize water, food, bedding, and other small objects. Improper packing of the autoclave load by stacking large quantities of material can likewise negatively affect autoclaving performance by limiting the penetration of hot steam. Ionizing-irradiation offers an alternative to moist heat sterilization. However, eradication of highly radioresistant bacteria such as *Deinococcus radiodurans*, which can withstand doses of 17.5 kGy γ-irradation, may present a challenge. Although rarely found and thus unlikely to occur in germ-free husbandries, contamination with *D. radiodurens* would require particularly high doses of ionizing-radiation (58, 82). Finally, thermolabile materials unsuitable for heat sterilization are treated with decontaminating gases such as ethylene oxide. Here, the application of unsuitable parameters in terms of exposure time, temperature, and humidity can interfere with the complete decimation of contaminants (82). In this regard, the cells and especially spores of the commonly found *Bacillus subtilis* are often employed to study or validate sterilization methods as they exhibit highly resistant properties (83, 84). Whereas decimal reduction of *S. faecalis* was achieved after 3 minutes exposition to ethylene oxide, the spore-forming *B. subtilis* survived twice as long (85). Interestingly, the age of cells and spores of *B. substilis* can significantly influence their resistance to sterilization using for instance heat or germicidal agents (86).

A study comparing commonly used liquid disinfectants discovered several bacteria originating from soil or plants, as well as *Micrococcus luteus*, in one of their contaminated gnotobiotic isolators. Since *M. luteus* is a bacterium found on human skin, it is possible that the contamination occurred through surfaces. By comparing chlorine-oxide- and peroxide-based disinfectants, they concluded that the latter was much less effective against vegetative bacteria and spores (70). Even though contaminations in germ-free husbandries are common issues, the identification of specific species is most likely not followed in every case due to cost and time limitations. Reports of fungal contaminants in particular are scarce and often left unspecified (e.g., mold). This is possibly attributed to the fact that fungi spread slowly and that contaminations are often not heavy enough to be detected through Gram staining. Certainly, concrete tracing of the source would help to improve internal standards and avoid errors that could introduce unwanted microorganisms in germ-free isolators. **Table 1** provides a list of bacterial contaminants that have been reported in germ-free husbandries or failed sterilization processes.

**Table 1:** Overview of described bacterial contaminants of germ-free isolators.

Phylum	Species	Reference
Proteobacteria	*Alcaligenes sp*	(59)
Firmicutes	*Bacillus licheniformis*	(70)
Firmicutes	*Bacillus subtilis*	(59)
Firmicutes	*Clostridium perfringens type D*	(77, 81)
Firmicutes	*Lactobacillus* sp.	(59)
Actinobacteria	*Micrococcus luteus*	(70)
Tenericutes	*Mycoplasma pulmonis*	(87)
Firmicutes	*Paenibacillus dendritiformis*	(70)
Firmicutes	*Paenibacillus macerans*	(70)
Firmicutes	*Paenibacillus motobuensis*	(70)
Firmicutes	*Paenibacillus thermophilus*	(70)
Ascomycota	*Penicillium* sp.	(59)
Firmicutes	*Sarcina* sp.	(59)
Firmicutes	*Staphylococcus aureus*	(59)
Firmicutes	*Staphylococcus epidermidis*	(59)
Firmicutes	*Turcibacter* sp.	(88)

Furthermore, in gnotobiotic experimentation, little attention has been paid to viral contamination, although infection of germ-free animals with viral particles were first described over 60 years ago (89–91). Virus-contaminated animals are often referenced as germ-free in the literature. Namely, leukaemia virus, mammary tumour virus, and lymphocytic choriomeningitis have been discovered and hypothesized to be transmitted from the maternal host to the fetus, a phenomenon termed congenital infection or vertical transmission (**Table 2**) (92). Given that specific viral contaminants can overcome the physical and immunological barrier of the placenta, special caution should be employed during screening of mice for derivation (93).

**Table 2:** Overview of described viral contaminants of germ-free mice.

Revtraviricetes	Leukaemia virus	(89)
Negarnaviricota	Lymphocytic choriomeningitis	(91)
Revtraviricetes	Mammary tumour virus	(89)

Vigilant working practice can be instrumental to detect contaminations at an early stage. Thereby, interventions may enable control of contaminations before these affect multiple colonies. In addition to sterility testing, regular observation of the animals’ appearance can provide information about their sterility status. Sickness, changes in fecal consistency or alterations of anatomical features such as a shrunken cecum or enlarged lymph nodes are potential indicators of microbial colonization of germ-free mice.

## 5 Methods applied for sterility testing

Monitoring sterility of gnotobiotic animal housing places high demands on sample preparation and testing methods. For various analyses such as microscopic examination, microbiological culture, or PCR-based evaluation, samples such as feces, fur, or urine can be utilized. Moreover, in order to ensure sterility within the isolator, many laboratories employ commercial or self-made swabs. Even though mice are held in individual cages, the whole isolator should be treated as a single sterile unit. As maintaining appropriate housing conditions involves changing bedding material, water, and enrichment inside the isolators, contamination is very likely to spread between all cages in a matter of days, depending on the frequency of handling. In this scenario, every cage could be seen as a sentinel cage and therefore, the collection of probes from only a few cages gives a sufficient approximation for the sterility state of the complete animal population inside an isolator. As the analyses are too elaborate to be performed directly inside an isolator, fresh samples need to be transported to the laboratory unaltered and analyzed directly, even if opening isolators to eject specimens also poses a potential threat of contamination. The time in between the probe collections can range from 24 hours to over a week or up to a month, depending on the planned experiments, housing conditions, and effort of the used techniques (33, 94). For instance, Nicklas et al. has suggested test bedding, food and fecal samples in the isolator every 4 weeks. Furthermore, for a more thorough examination of the organs, necropsy can be performed once every 3 to 6 months using various examination methods (58). Regular testing enables weekly monitoring of isolator sterility and facilitates early detection of contamination at its initial stages. However, the risk of contamination can increase due to frequent transfer of testing materials and samples.

In murine gut microbiota, the abundance of strict anaerobes exceeds facultative anaerobes or aerobes by a factor of 100 to 1000 (95). Therefore, the viability of most gut microbes is strongly impaired in the presence of oxygen (96). In principle, it is possible to cultivate anaerobic bacteria from feces in hypoxic chambers (97). On their way into a sterile isolator, contaminants most likely experience exposure to oxygen, so they predominantly might be characterized as aerobic or at least aerotolerant. This would be in line with bacteria from the genera *Staphylococcus*, *Acinetobacter*, *E. coli*, *Pseudomonas*, *Klebsiella*, and *Aspergillus*, being commonly found on surfaces (98–101). One exception might be anaerobic bacteria of the genus *Clostridium*, that can survive exposure to atmospheric oxygen and even temperatures up to 121°C by forming spores (102–105).

### 5.1 Probe sampling

When screening for contamination in isolators, swabs and fecal collection are the most frequent methods. The treatment of the samples prior to analysis may affect the results, the conditions during transport, storage, and analysis should mirror those during probe sampling. For this, primary sterile materials as well as swabs and smears should be kept at room temperature (106).

Swabs are used to collect material from isolator and cage surfaces. All-in-one systems like Isolator and Clean Room (ICR)-swabs already contain nutrient broth medium to cultivate contaminants sticking to the tip of the swab. They are irradiated with doses ranging between 25 kGy and 35 kGy and allow growth of contaminants while avoiding unwanted contaminations during further processing (107). ICR-swabs with soyabean casein digest medium have been reported to detect several microorganisms found in aseptic environments like *Staphylococcus aureus, Pseudomonas aeruginosa, Candida albicans, Aspergillus brasiliensis, Bacillus subtilis, Micrococcus luteus, Staphylococcus capitis* and *Bacillus pumilis* when inoculated with less than 10 colony forming units (CFU) of the respective microorganism (108).

While swabs are applicable to sample surfaces inside an isolator, analysis of feces allows the assessment of sterility of the housed animals directly. Fecal samples can be taken either as excreted pellets from within the cage or by dissection after sacrificing the mouse. While the latter minimizes the risk of false positive testing results if the dissection is performed in a sterile environment, it is not applicable for continuous testing of isolators. For this, collection of fecal pellets as fresh as possible with sterile instruments is more sustainable. Importantly, the storage conditions of fecal samples after collection can affect microbial content. Samples that are kept at room temperature for up to 72 h can display time-dependent changes in the composition of microbes, like an increase in the relative abundances of *Bifidobacterium* species as well as a decrease in the relative abundances of *Anaerostipes*, *Ruminococcus*, *Faecalibacterium* and *Lachnospiraceae* species (109, 110). If testing is not performed immediately after sampling, storage at -80°C can retain the microbial information, at least for DNA-based analyses, for a longer period of time with only minor changes in bacterial abundances, even after two years (111).

### 5.2 Testing methods

Fecal sample testing can give information both on the presence of living bacteria and bacteria-borne products. While the first entails microscopic examination and culturing bacteria in liquid or solid medium, the latter is accomplished by amplification and/or sequencing methods. Both approaches have their drawbacks as well as advantages that are described in more detail below. Of note, serological and fungal testing of germ-free mouse colonies is also required.

### 5.3 Microscopic examination

Gross observation and microscopic examination of sample material from germ-free isolators can provide evidence of sterility status and potential contaminants, but requires an experienced microscopist. Fecal and blood smears, gastrointestinal (GI) contents, accumulated waste, and organ imprints have been suggested as sample materials. Gram staining has been characterized as a method for broad observation and detection of bacteria or fungi, as it allows the detection of Gram-positive and Gram-negative bacterial species based on the stain of their cell walls. Gram-positive bacteria present a thicker peptidoglycan layer and stain violet, while Gram-negative bacteria stain red due to the thinner peptidoglycan layer in their cell wall (112). Heidenhain’s Iron Alum Hematoxylin (HIAH)-, Machiavello-, Giesma, and Kinyoun’s acid-fast stain could be used for specific detection of protozoa, Rickettsiae, Bartonellaceae and mycobacteria, respectively (**Table 3**). However, many bacterial strains are sensitive to Gram staining or culture and hence difficult to identify using standard techniques.

**Table 3:** Methods for detection of contaminants.

Sample collection	Methods	Detection
**Stained fresh fecal smear and GI contents**	Gram stain	Bacteria and fungi
	HIAH stain	Protozoa
	HIAH stain after zinc sulphate flotation	Protozoan cysts
**GI contents**	Machiavello stain	Rickettsiae
	Kinyoun’s acid-fast stain	Mycobacteria
**Accumulated waste (feces, urine, diet, water)**	Gram stain	Bacteria and fungi
**Blood smears and organ imprints**	Giemsa stain	Protozoa and Bartonellaceae
	Machiavello stain	Rickettsiae
	Kinyoun’s acid-fast stain	Mycobacteria
**Fecal or cecal contents, organs and tissues**	Wet mounts (direct wet mounts, after concentration with flotation, pressure plates of tissues and organs, sediment digestion in gastric juice) Histopathological examination by H&E staining	Motile and nonmotile bacterial forms, mycological forms, protozoa, helminths

### 5.4 Microbiological culture

Microbiological culture in liquid or solid media can be used to detect contamination of living bacteria in a gnotobiotic facility. Fluid thioglycollate medium (FTM) and soybean-casein digest medium (SCDM) are currently suggested to be the most appropriate liquid media for sterility testing. While FTM may identify both aerobic and anaerobic species, SCDM is mostly used to detect fungi and aerobic bacteria. The most widely used solid medium for sterility controls is brain-heart infusion agar with 5% sterile defibrinated horse or goat blood (113). Sabouraud’s dextrose medium is recommended to rapidly detect fungal contamination, though other media also support fungal growth (114).

**Table 4** provides examples of media commonly used in microbiological culturing methods. Brain-heart Infusion Broth or agar, FTM, SCDM as well as growth and ascorbic acid media, are mainly used to identify generic contaminants. However, if the objective is to target a specific bacterial strain, selective culture media should be utilized. For instance, MacConkey medium is specifically designed to target Gram-negative and enteric bacteria, based on their ability to ferment lactose and utilize bile salts. Lactose-fermenting bacteria like *E. coli*, *Klebsiella*, and *Enterobacter* produce acids that results in pink colonies (122). Similarly, the tryptone and yeast extract-rich Luria-Bertani medium is widely used for detection and cultivation of *E. coli* (121). Brucella agar serves the purpose of targeting not only *Brucella* species but also anaerobic gut residents such as *Bacteroides* and *Prevotella* species (119). Additionally, reinforced clostridial medium allows the specific detection of *Clostridium* sp. For the general detection of anaerobic species, anaerobic blood agar can be applied (115). Combining different culture methods and media allows the detection of bacteria that require specific growth conditions.

**Table 4:** Microbiological culture media.

Media	Target	Conditions	Reference
**Anaerobic blood agar**	All-purpose	Anaerobic	(115)
**Ascorbic acid medium**	All-purpose	Aerobic and anaerobic	(116, 117)
**Brain-heart infusion medium**	All-purpose, Streptococcus and Neisseria	Aerobic and anaerobic	(118)
**Brucella agar**	*Brucella* sp.*, Bacteroides* and *Prevotella* sp.	Anaerobic	(119)
**Fluid thioglycolate medium**	All-purpose	Aerobic and anaerobic	(120)
**Luria-Bertani medium**	*E. coli* and fast-growing bacteria	Aerobic	(121)
**MacConkey medium**	Gram-negative bacteria	Aerobic	(122, 123)
**Growth medium**	All-purpose	Aerobic and anaerobic	(124, 125)
**Reinforced clostridial medium**	*Clostridium* sp.	Anaerobic	(126)
**Sabourad’s dextrose medium**	Fungi/yeast/molds	Aerobic	(114)
**Soybean-casein digest medium**	All-purpose, fungi	Aerobic and anaerobic	(127)

For growth analyses, collected fecal samples or cecal content can be dissolved in media either under aerobic or anaerobic conditions. Temperatures of 25°C, 37°C, and 55°C have been recommended for sample incubation. The incubation time period for microbiological cultures varies based on the typical growth characteristics of the bacteria being cultured. Fast-growing bacteria, like *E. coli*, can form visible colonies within 24 hours of incubation (128). However, other bacteria with slower growth rates may require 48 hours or longer to develop visible colonies (129, 130). Moreover, fungi and yeast often require several days or weeks to display visible growth (131). Therefore, it is crucial to consider specific growth requirements, such as temperature, oxygen, and incubation ´periods for the targeted microorganism.

**Figure 2:**
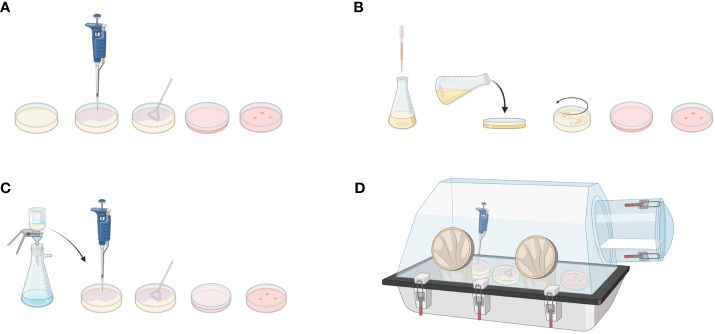
Microbiological culturing methods. Spread plate method: **(A)** Diluted sample is applied to the solid agar using Drigalski spatula. After incubation, bacterial colonies can be counted on the plate surface. **(B)** Pour plate method: Diluted sample is mixed with a melted agar medium and poured into plate. After incubation, bacteria are visible in the agar. **(C)** Filtration method: Diluted sample is passed through a filter. Bacteria attached to the filter are applied to the agar plate for further incubation and evaluation. **(D)** Anerobic culture method: To detect anaerobic bacterial species, spread plate method can be applied in the anaerobic chamber. Created with BioRender.com.

The most common method of culturing microorganisms is the spread plate method. In this method, a sterile Drigalski spatula is used to spread the diluted sample over a solid agar surface. After incubation, colonies growing on the surface of the medium can be counted and identified (**Figure 2A**). Alternatively, the diluted sample can be mixed with melted agar and poured into a sterile Petri dish (**Figure 2B**). Filtration involves passing a sample through a filter and collecting bacteria on its surface. Filtered bacteria can then be collected and placed on the surface of a blood agar plate for subsequent incubation (**Figure 2C**). To identify anaerobic bacterial species, samples can be spread over agar plates and incubated in anaerobic chambers or jars (**Figure 2D**).

In conclusion, gross observation, microbiological culture, and microscopic examination are commonly utilized to detect contamination within 3-5 days. The advantages of these systems are their ease of use, efficiency, and low cost of materials. However, detection of stained bacteria can be challenging and result in a false-positive or -negative outcome.

### 5.5 PCR-based methods

Polymerase chain reaction (PCR)–based methods can be used to identify bacterial contamination in germ-free facilities (132). Packey et al. have provided a comprehensive description of PCR-based methods, including random amplification of polymorphic DNA (RAPD) PCR, PCR detection of the 16S rRNA gene, and qPCR, as well as 16S rRNA sequencing, suggesting their use in screening germ-free isolators for bacterial contamination. Compared to traditional methods such as Gram-staining and culture, PCR-based approaches are deemed more sensitive and suitable for detecting contaminants (133). However, despite their advantages, PCR-based methods also have potential drawbacks. For example, they can yield false-positive or false-negative results, have a limited scope of detection, and are expensive. Also, PCR conditions need to be carefully optimized to selectively target the bacterial species of interest, requiring technical expertise, training, and expensive equipment and materials (134).

There is a wealth of different universal primer sets described in the literature to detect the bacterial 16S rRNA-gene by PCR. A commonly used universal primer set to detect the bacterial 16S rRNA-gene is (133):

Forward: UniF: 5′-GTGSTGCAYGGYTGTCGTCA-3′
Reverse: UniR: 5′-ACGTCRTCCMCACCTTCCTC-3′

Interestingly, Fontaine et al. (2015) conducted a study comparing Gram-staining and culture to molecular PCR-based screening, concluding that none of the screening assays was able to detect fewer than 105 CFU/g of feces (88). Unlike the earlier study by Packey et al., they were able to quantify the limits of PCR detection and directly compare traditional and PCR-based screening methods (133). Therefore, the study concluded that both screening methods were suitable for detecting contamination, without a clear advantage of PCR-based methods over microscopic screening methods. The culturing method was found to be useful for rapidly detecting contamination, while PCR-based methods could be used to confirm the origin of contamination. However, many sterilized rodent diets may still contain small quantities of bacterial 16S rDNA, which hampers PCR-based detection of isolator contamination.

Taken together, Gram staining of fecal content and bacterial culture have remained proven methods for bacterial and fungal detection in germ-free facilities for over 80 years, while PCR-based approaches allow exact detection of specific and targeted bacterial species. In conclusion, it is advisable to use microbiological culturing methods for regular testing in germ-free isolators, while PCR-based screening assays can be employed to determine the specific species responsible for the contamination.

### 5.6 Serological testing

Serological testing is crucial in gnotobiotic facilities because some contaminants, like viruses, cannot be easily detected through traditional culturing methods. Monitoring for specific viruses such as mouse hepatic virus (MHV) and mouse parvovirus, as discussed in the study by Brielmeier et al., is essential (135). Parvoviruses, despite being highly contagious, may progress asymptomatically, making their detection challenging. MHV, on the other hand, can lead to respiratory and enteric diseases in mice (136, 137). Similarly, common murine viruses like Sendai virus (SV) and murine norovirus (MNV) can cause illnesses in mice and potentially influence research outcomes (138–140). Thus, diligent monitoring and testing for these contaminants are crucial for maintaining the health and reliability of experiments in gnotobiotic facilities. Viral contaminations can be detected using ELISA, immunofluorescence assay, or PCR.

### 5.7 Testing for fungal contamination

Fungal contamination is typically detected using culturing methods, which are time-consuming and can take from 5 days to 2 weeks. However, there are faster methods available for detecting fungal contamination. One such method involves using an electronic nose (e-nose), which can detect fungi even before their spores are recognizable. E-nose technology analyzes volatile organic compounds (VOCs) produced by fungi (141, 142). This technology, previously applied in food and medical diagnostics, can be valuable in gnotobiotic facilities.

Another rapid identification method involves matrix-assisted laser desorption/ionization time-of-flight mass spectrometry (MALDI-TOF MS), which allows for the targeted and quick identification of mycobacteria and molds. This technology has been developed and optimized over the last 35 years and is widely used in microbiology not only for detection, but also for precise identification of fungal and bacterial species (143). However, it is important to note that these advanced methods can be expensive in comparison to traditional culturing methods, which are easy to perform and do not require special materials or kits.

### 5.8 Experiences on testing protocol for bacterial and fungal contaminants

**Figure 3:**
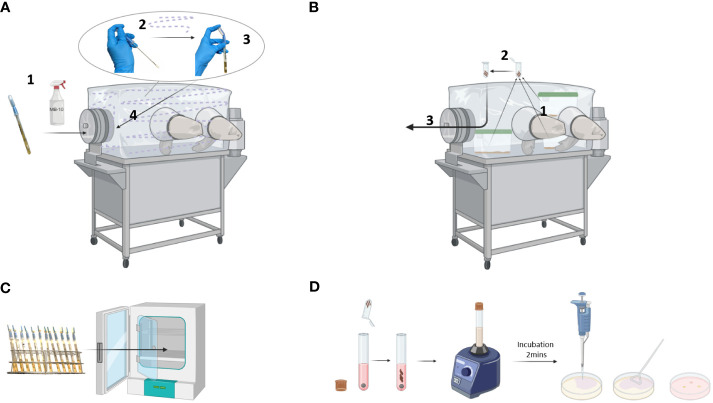
Sterility control of a gnotobiotic isolator. **(A)** ICR swab sampling: ICR swab was incubated with germicide for 1 hour in the isolator transfer port (1). Then, the swab was opened and used to sample the walls, gloves, and connecting parts of the isolator (2). Once the swab was placed into a tube, the reservoir containing the culture medium was squeezed to completely cover the swab (3) before transferring it out of the isolator (4). **(B)** Fecal samples: Fecal pellets were collected from 3 randomly chosen cages (1) and placed into a reaction tube (2). The tube was then transferred out of the isolator (3). **(C)** Incubation: ICR swabs were incubated at 37°C for 3 days. **(D)** Microbiological testing: The fecal samples were dissolved in culture medium by vortexing, incubated for 2 minutes, and applied to solid agar using a Drigalski spatula. Created with BioRender.com.

Protocol for a weekly sterility control of gnotobiotic isolators:

1. Place the sterile ICR swabs in the isolator transfer port, and spray them with germicide and incubate for 1 hour. After incubation, transfer the swab inside the isolator. Perform the swabbing as depicted in **Figure 3A**.
2. Collect 3 fecal pellets from 3 random cages and place them in autoclaved 2 ml reaction tubes (**Figure 3B**).
3. Transfer the ICR swabs and fecal samples outside the isolator and transport them immediately to the lab for further analysis.
4. Incubate the ICR swabs upright at 37°C for 3 days, with no shaking (**Figure 3C**).
5. In a sterile laminar flow hood, open the reaction tubes with fecal samples. Transfer the feces into 14 ml culture tubes, also adding one sterile stainless-steel bead (**Figure 3D**).
6. Add 5 ml of Brain-Heart (BH) Infusion medium to the samples and vigorously vortex for 5 minutes.
7. Prepare a negative control by adding 5 ml of BH medium to stored feces material from germ-free (GF) mice. Prepare a positive control by dissolving feces from CONV-R mice in 5 ml of BH medium.
8. After 2 minutes of incubation, apply 100 µl of supernatant to a blood agar plate containing 5% goat blood. Spread the sample evenly using a Drigalski spatula.
9. Incubate the blood agar plates upside down at 37°C for 5 days.

**Figure 4:**
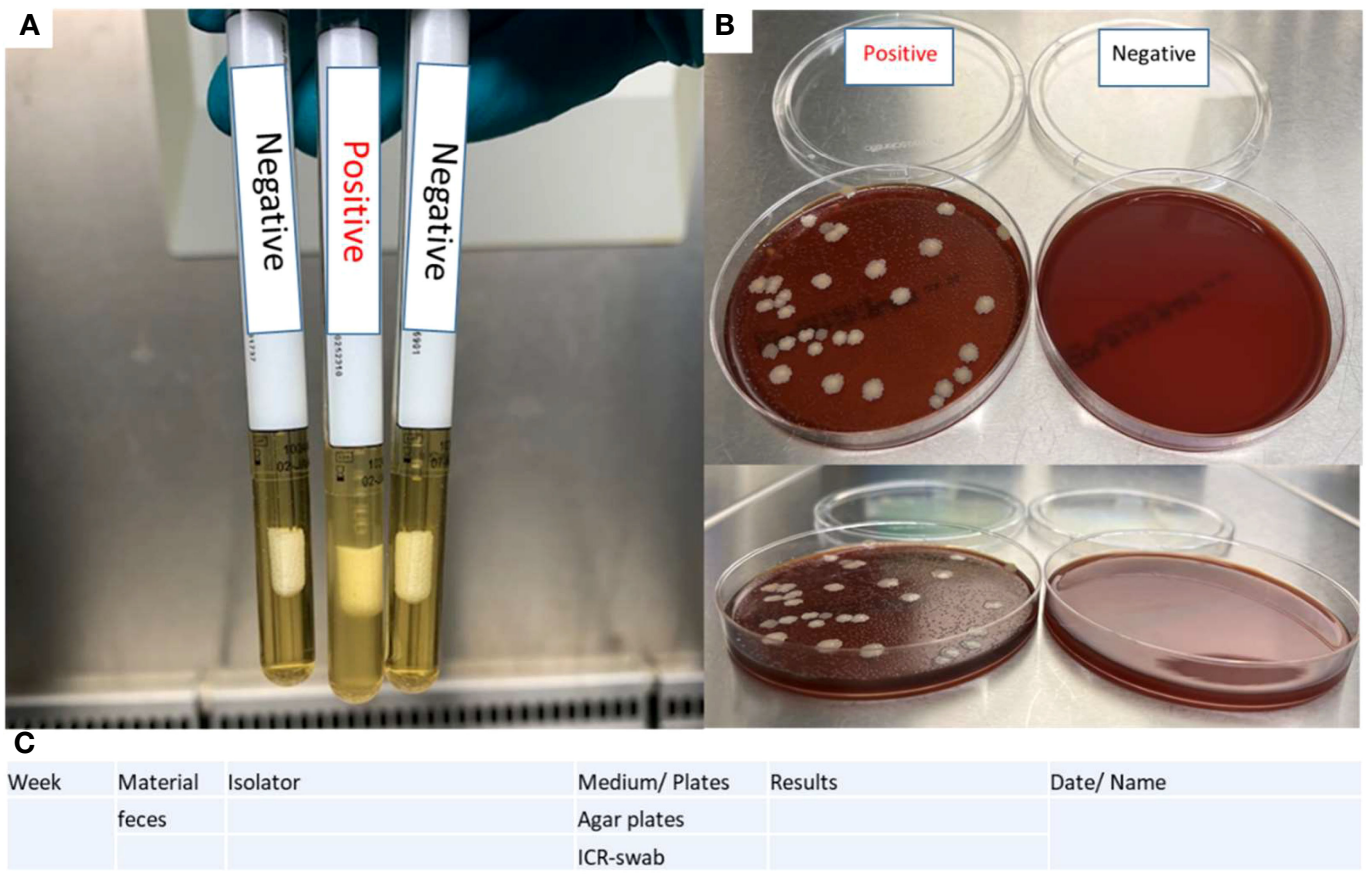
Evaluation of sterility tests. **(A)** ICR swab turbidity test determines the positivity based on the presence of a turbid medium on the swab. **(B)** Blood agar plates contain positive controls (feces of CONV-R mice) and negative controls (BH medium). **(C)** A documentation sheet is provided for sterility testing.

Evaluation:

1. Shake the ICR swabs before visual examination and documentation. Place them in a metal rack and document them as shown in **Figure 4A**. A photograph is taken with a suitable background and lighting conditions in the sterile laminar flow hood.
➢ Swabs in turbid medium should be considered positive.
2. Place the blood agar plates in the fume cupboard in an open position as depicted in **Figure 4B**.
➢ If bacterial colonies are visible on the surface of the blood agar plates, consider the corresponding isolator positive.
3. Enter results in a documentation sheet (**Figure 4C**)

In case of any detected contamination, repeat the sample examination to exclude false-positive outcomes caused by contamination during transportation or probe preparation.

## 6 Conclusions

Standardized fortnightly sterility testing of individual mouse isolator units is mandatory to ensure the germ-free status of axenic mouse colonies. Since diet is a possible source of 16S rRNA gene contaminants that do not necessarily stem from living microbes, but could be remainders of irradiation or heat-inactivated microbes, microbial culturing is essential and a more reliable method to detect possible contaminations of germ-free mouse colonies. Although some diets may allow for sterility controls based on the PCR amplification of conserved regions in the 16S rRNA gene using universal primers, ICR swabs and blood agar cultures are reliable sterility testing methods to monitor germ-free housing.

## Author contributions

CR: Conceptualization, Supervision, Writing – original draft, Writing – review & editing. OD: Writing – original draft, Writing – review & editing. MM: Writing – original draft, Writing – review & editing. NP: Writing – original draft, Writing – review & editing. MK: Writing – original draft, Writing – review & editing. ZG: Writing – original draft, Writing – review & editing. NS: Writing – review & editing. AMe: Writing – review & editing. YB: Writing – review & editing. VR: Writing – review & editing. AMa: Writing – original draft, Writing – review & editing. GP: Writing – review & editing. KK: Writing – original draft, Writing – review & editing. MB: Writing – review & editing. LPG: Writing – review & editing.

## References

[1] Berg G Rybakova D Fischer D Cernava T Vergès M-CC Charles T. Microbiome definition re-visited: old concepts and new challenges. Microbiome (2020) 8:103. doi: 10.1186/s40168-020-00875-0 32605663 PMC7329523

[2] Esser D Lange J Marinos G Sieber M Best L Prasse D. Functions of the microbiota for the physiology of animal metaorganisms. J Innate Immun (2019) 11:393–404. doi: 10.1159/000495115 30566939 PMC6738199

[3] Johnson EL Heaver SL Waters JL Kim BI Bretin A Goodman AL. Sphingolipids produced by gut bacteria enter host metabolic pathways impacting ceramide levels. Nat Commun (2020) 11:2471. doi: 10.1038/s41467-020-16274-w 32424203 PMC7235224

[4] Reinhardt C Bergentall M Greiner TU Schaffner F Ostergren-Lundén G Petersen LC. Tissue factor and PAR1 promote microbiota-induced intestinal vascular remodelling. Nature (2012) 483:627–31. doi: 10.1038/nature10893 PMC3885420 22407318

[5] Schaupp L Muth S Rogell L Kofoed-Branzk M Melchior F Lienenklaus S. Microbiota-induced type I interferons instruct a poised basal state of dendritic cells. Cell (2020) 181:1080–1096.e19. doi: 10.1016/j.cell.2020.04.022 32380006

[6] Fransen F van Beek AA Borghuis T Aidy SE Hugenholtz F van der Gaast-de Jongh C. Aged gut microbiota contributes to systemical inflammaging after transfer to germ-free mice. Front Immunol (2017) 8:1385. doi: 10.3389/fimmu.2017.01385 29163474 PMC5674680

[7] Formes H Bernardes JP Mann A Bayer F Pontarollo G Kiouptsi K. The gut microbiota instructs the hepatic endothelial cell transcriptome. iScience (2021) 24:103092. doi: 10.1016/j.isci.2021.103092 34622147 PMC8479694

[8] Lee KA Thomas AM Bolte LA Björk JR de Ruijter LK Armanini F. Cross-cohort gut microbiome associations with immune checkpoint inhibitor response in advanced melanoma. Nat Med (2022) 28:535–44. doi: 10.1038/s41591-022-01695-5 PMC8938272 35228751

[9] Vieira-Silva S Falony G Belda E Nielsen T Aron-Wisnewsky J Chakaroun R. Statin therapy is associated with lower prevalence of gut microbiota dysbiosis. Nature (2020) 581:310–5. doi: 10.1038/s41586-020-2269-x 32433607

[10] Smith K McCoy KD Macpherson AJ. Use of axenic animals in studying the adaptation of mammals to their commensal intestinal microbiota. Semin Immunol (2007) 19:59–69. doi: 10.1016/j.smim.2006.10.002 17118672

[11] Basic M Bleich A. Gnotobiotics: past, present and future. Lab Anim (2019) 53:232–43. doi: 10.1177/0023677219836715 31096878

[12] Human microbiome project consortium. Structure, function and diversity of the healthy human microbiome. Nature (2012) 486:207–14. doi: 10.1038/nature11234 PMC3564958 22699609

[13] Derrien M Alvarez A-S de Vos WM. The gut microbiota in the first decade of life. Trends Microbiol (2019) 27:997–1010. doi: 10.1016/j.tim.2019.08.001 31474424

[14] Bayer F Dremova O Khuu MP Mammadova K Pontarollo G Kiouptsi K. The interplay between nutrition, innate immunity, and the commensal microbiota in adaptive intestinal morphogenesis. Nutrients (2021) 13:2198. doi: 10.3390/nu13072198 34206809 PMC8308283

[15] Bolsega S Bleich A Basic M. Synthetic microbiomes on the rise-application in deciphering the role of microbes in host health and disease. Nutrients (2021) 13:4173. doi: 10.3390/nu13114173 34836426 PMC8621464

[16] Falk PG Hooper LV Midtvedt T Gordon JI. Creating and maintaining the gastrointestinal ecosystem: what we know and need to know from gnotobiology. Microbiol Mol Biol Rev (1998) 62:1157–70. doi: 10.1128/MMBR.62.4.1157-1170.1998 PMC98942 9841668

[17] Haas BJ Gevers D Earl AM Feldgarden M Ward DV Giannoukos G. Chimeric 16S rRNA sequence formation and detection in Sanger and 454-pyrosequenced PCR amplicons. Genome Res (2011) 21:494–504. doi: 10.1101/gr.112730.110 21212162 PMC3044863

[18] Schloss PD Hay AG Wilson DB Gossett JM Walker LP. Quantifying bacterial population dynamics in compost using 16S rRNA gene probes. Appl Microbiol Biotechnol (2005) 66:457–63. doi: 10.1007/s00253-004-1727-y 15368083

[19] Rausch P Basic M Batra A Bischoff SC Blaut M Clavel T. Analysis of factors contributing to variation in the C57BL/6J fecal microbiota across German animal facilities. Int J Med Microbiol (2016) 306:343–55. doi: 10.1016/j.ijmm.2016.03.004 27053239

[20] Münzel T Sørensen M Lelieveld J Hahad O Al-Kindi S Nieuwenhuijsen M. Heart healthy cities: genetics loads the gun but the environment pulls the trigger. Eur Heart J (2021) 42:2422–38. doi: 10.1093/eurheartj/ehab235 PMC8248996 34005032

[21] Rothschild D Weissbrod O Barkan E Kurilshikov A Korem T Zeevi D. Environment dominates over host genetics in shaping human gut microbiota. Nature (2018) 555:210–5. doi: 10.1038/nature25973 29489753

[22] Sellon RK Tonkonogy S Schultz M Dieleman LA Grenther W Balish E. Resident enteric bacteria are necessary for development of spontaneous colitis and immune system activation in interleukin-10-deficient mice. Infect Immun (1998) 66:5224–31. doi: 10.1128/IAI.66.11.5224-5231.1998 PMC108652 9784526

[23] Bayer F Ascher S Pontarollo G Reinhardt C. Antibiotic treatment protocols and germ-free mouse models in vascular research. Front Immunol (2019) 10:2174. doi: 10.3389/fimmu.2019.02174 31572384 PMC6751252

[24] Nuttall GHF Thierfelder H. Thierisches Leben ohne Bakterien im Verdauungskanal. Zeitschrift für Physiologische Chemie (1896) 21:109–21. doi: 10.1515/bchm2.1896.21.2-3.109

[25] Küster F ed. Die Gewinnung, Haltung und Aufzucht keimfreier Tiere und ihre Bedeutung für die Erforschung natürlicher Lebensvorgänge. 1st ed. Berlin, Heidelberg: Springer (1914). doi: 10.1007/978-3-662-26235-1_1

[26] Glimstedt G ed. Bakterienfreie Meerschweinchen: Aufzucht, Lebensfähigkeit u. Wachstum, nebst Untersuchgn über das lymphatische Gewebe. Lund: Hakan Ohlsson/Kobenhavn: Levin & Munksgaard (1936).

[27] Reyniers JA. Germ-free life applied to nutrition studies. Lobund Rep (1946) 1):87–120. 20247585

[28] Pleasants JR. Rearing germfree cesarean-born rats, mice, and rabbits through weaning. Ann N Y Acad Sci (1959) 78:116–26. doi: 10.1111/j.1749-6632.1959.tb53099.x 14433463

[29] Gustafsson B. Germ-free rearing of rats. Acta Anat (Basel) (1946) 2:376–91. doi: 10.1159/000140222 20256962

[30] Gustafsson BE. Lightweight stainless steel systems for rearing germfree animals. Ann N Y Acad Sci (1959) 78:17–28. doi: 10.1111/j.1749-6632.1959.tb53092.x 13830425

[31] Trexler PC Reynolds LI. Flexible film apparatus for the rearing and use of germfree animals. Appl Microbiol (1957) 5:406–12. doi: 10.1128/am.5.6.406-412.1957 PMC1057339 13488447

[32] Hecht G Bar-Nathan C Milite G Alon I Moshe Y Greenfeld L. A simple cage-autonomous method for the maintenance of the barrier status of germ-free mice during experimentation. Lab Anim (2014) 48:292–7. doi: 10.1177/0023677214544728 25097255

[33] Basic M Bolsega S Smoczek A Gläsner J Hiergeist A Eberl C. Monitoring and contamination incidence of gnotobiotic experiments performed in microisolator cages. Int J Med Microbiol (2021) 311:151482. doi: 10.1016/j.ijmm.2021.151482 33636479

[34] Inzunza J Midtvedt T Fartoo M Norin E Osterlund E Persson A-K. Germfree status of mice obtained by embryo transfer in an isolator environment. Lab Anim (2005) 39:421–7. doi: 10.1258/002367705774286439 16197709

[35] Okamoto M Matsumoto T. Production of germfree mice by embryo transfer. Exp Anim (1999) 48:59–62. doi: 10.1538/expanim.48.59 10067209

[36] Reyniers JA Sacksteder MR. Apparatus and method for shipping germ-free and disease-free animals via public transportation. Appl Microbiol (1958) 6:146–52. doi: 10.1128/am.6.2.146-152.1958 PMC1057374 13521940

[37] Mahowald MA Rey FE Seedorf H Turnbaugh PJ Fulton RS Wollam A. Characterizing a model human gut microbiota composed of members of its two dominant bacterial phyla. Proc Natl Acad Sci U S A (2009) 106:5859–64. doi: 10.1073/pnas.0901529106 PMC2660063 19321416

[38] Dahlgren UI Wold AE Hanson LA Midtvedt T. The secretory antibody response in milk and bile against fimbriae and LPS in rats monocolonized or immunized in the Peyer’s patches with Escherichia coli. Immunology (1990) 71:295–300. 1977693 PMC1384319

[39] Rask C Evertsson S Telemo E Wold AE. A full flora, but not monocolonization by Escherichia coli or lactobacilli, supports tolerogenic processing of a fed antigen. Scand J Immunol (2005) 61:529–35. doi: 10.1111/j.1365-3083.2005.01598.x 15963047

[40] Gillilland MG Erb-Downward JR Bassis CM Shen MC Toews GB Young VB. Ecological succession of bacterial communities during conventionalization of germ-free mice. Appl Environ Microbiol (2012) 78:2359–66. doi: 10.1128/AEM.05239-11 PMC3302583 22286988

[41] Clavel T Gomes-Neto JC Lagkouvardos I Ramer-Tait AE. Deciphering interactions between the gut microbiota and the immune system via microbial cultivation and minimal microbiomes. Immunol Rev (2017) 279:8–22. doi: 10.1111/imr.12578 28856739 PMC5657458

[42] Dewhirst FE Chien CC Paster BJ Ericson RL Orcutt RP Schauer DB. Phylogeny of the defined murine microbiota: altered Schaedler flora. Appl Environ Microbiol (1999) 65:3287–92. doi: 10.1128/AEM.65.8.3287-3292.1999 PMC91493 10427008

[43] Schaedler RW Dubos R Costello R. The development of the bacterial flora in the gastrointestinal tract of mice. J Exp Med (1965) 122:59–66. doi: 10.1084/jem.122.1.59 14325473 PMC2138024

[44] Stehr M Greweling MC Tischer S Singh M Blöcker H Monner DA. Charles river altered schaedler flora (CRASF) remained stable for four years in a mouse colony housed in individually ventilated cages. Lab Anim (2009) 43:362–70. doi: 10.1258/la.2009.0080075 19535393

[45] Gomes-Neto JC Mantz S Held K Sinha R Segura Munoz RR Schmaltz R. A real-time PCR assay for accurate quantification of the individual members of the altered schaedler flora microbiota in gnotobiotic mice. J Microbiol Methods (2017) 135:52–62. doi: 10.1016/j.mimet.2017.02.003 28189782 PMC5365401

[46] Elie C Mathieu A Saliou A Villain A Darnaud M Leulier F. Draft genome sequences of 15 bacterial species constituting the stable defined intestinal microbiota of the GM15 gnotobiotic mouse model. Microbiol Resour Announc (2020) 9:e00686–20. doi: 10.1128/MRA.00686-20 PMC7453283 32855247

[47] Eberl C Ring D Münch PC Beutler M Basic M Slack EC. Reproducible colonization of germ-free mice with the oligo-mouse-microbiota in different animal facilities. Front Microbiol (2019) 10:2999. doi: 10.3389/fmicb.2019.02999 31998276 PMC6965490

[48] Becker N Kunath J Loh G Blaut M. Human intestinal microbiota: characterization of a simplified and stable gnotobiotic rat model. Gut Microbes (2011) 2:25–33. doi: 10.4161/gmic.2.1.14651 21637015

[49] Kovatcheva-Datchary P Shoaie S Lee S Wahlström A Nookaew I Hallen A. Simplified intestinal microbiota to study microbe-diet-host interactions in a mouse model. Cell Rep (2019) 26:3772–3783.e6. doi: 10.1016/j.celrep.2019.02.090 30917328 PMC6444000

[50] Licht TR Madsen B Wilcks A. Selection of bacteria originating from a human intestinal microbiota in the gut of previously germ-free rats. FEMS Microbiol Lett (2007) 277:205–9. doi: 10.1111/j.1574-6968.2007.00962.x 18031341

[51] Wos-Oxley M Bleich A Oxley APA Kahl S Janus LM Smoczek A. Comparative evaluation of establishing a human gut microbial community within rodent models. Gut Microbes (2012) 3:234–49. doi: 10.4161/gmic.19934 PMC3427216 22572831

[52] Pang X Hua X Yang Q Ding D Che C Cui L. Inter-species transplantation of gut microbiota from human to pigs. ISME J (2007) 1:156–62. doi: 10.1038/ismej.2007.23 18043625

[53] Goodman AL Kallstrom G Faith JJ Reyes A Moore A Dantas G. Extensive personal human gut microbiota culture collections characterized and manipulated in gnotobiotic mice. Proc Natl Acad Sci U S A (2011) 108:6252–7. doi: 10.1073/pnas.1102938108 PMC3076821 21436049

[54] Nguyen TLA Vieira-Silva S Liston A Raes J. How informative is the mouse for human gut microbiota research? Dis Model Mech (2015) 8:1–16. doi: 10.1242/dmm.017400 25561744 PMC4283646

[55] Ridaura VK Faith JJ Rey FE Cheng J Duncan AE Kau AL. Gut microbiota from twins discordant for obesity modulate metabolism in mice. Science (2013) 341:1241214. doi: 10.1126/science.1241214 24009397 PMC3829625

[56] Kennedy EA King KY Baldridge MT. Mouse microbiota models: comparing germ-free mice and antibiotics treatment as tools for modifying gut bacteria. Front Physiol (2018) 9:1534. doi: 10.3389/fphys.2018.01534 30429801 PMC6220354

[57] Germfree vertebrates: present status. Annals of the New York Academy of Science. Am J Public Health Nations Health (1959) 49:1569. doi: 10.2105/AJPH.49.11.1569-b

[58] Nicklas W Keubler L Bleich A. Maintaining and monitoring the defined microbiota status of gnotobiotic rodents. ILAR J (2015) 56:241–9. doi: 10.1093/ilar/ilv029 26323633

[59] Reyniers JA Trexler PC Ervin RF. Rearing germ-free albino rats. Lobound Rep (1946) 1:1–84. 20247584

[60] Vowles C Anderson N Eaton K eds. Gnotobiotic Mouse Technology: An Illustrated Guide. 1st ed. Boca Raton: CRC Press (2016).

[61] Hedrich H ed. The laboratory mouse. 2nd ed. Amsterdam: Elsevier Academic Press (2012).

[62] Fox JG Davisson MT Quimby FW Barthold SW Newcomer CE Smith AL eds. The mouse in Biomedical Research. 2nd ed. Cambridge, Massachusetts: Academic Ress (2007).

[63] Makin TJ Tzipori S. Inexpensive techniques for the production and maintenance of gnotobiotic piglets, calves and lambs. Aust Vet J (1980) 56:353–8. doi: 10.1111/j.1751-0813.1980.tb09558.x 6449193

[64] Meyer RC Bohl EH Kohler EM. Procurement and maintenance of germ-free seine for microbiological investigations. Appl Microbiol (1964) 12:295–300. doi: 10.1128/am.12.4.295-300.1964 14199016 PMC1058120

[65] Paik J Pershutkina O Meeker S Yi JJ Dowling S Hsu C. Potential for using a hermetically-sealed, positive-pressured isocage system for studies involving germ-free mice outside a flexible-film isolator. Gut Microbes (2015) 6:255–65. doi: 10.1080/19490976.2015.1064576 PMC4615381 26177210

[66] Scatina J Abdel-Rahman MS Gerges SE Khan MY Gona O. Pharmacodynamics of alcide, a new antimicrobial compound, in rat and rabbit. Fundam Appl Toxicol (1984) 4:479–84. doi: 10.1016/0272-0590(84)90206-9 6745537

[67] Pell-Walpole C Waller M. Effective sterilization of a plastic film rack isolator with “Alcide. ” Lab Anim (1984) 18:349–50. doi: 10.1258/002367784780865333 6513500

[68] Newman LE Whitehair CK Mullaney TP. Gnotobiotic calves: derivation, maintenance, thiry-vella loop preparation, and ethylene oxide gas sterilization. Am J Vet Res (1986) 47:2632–6. 3800124

[69] Herbert J Roser B. Simple methods which maintain the barrier status of specific-pathogen-free animals during experimentation. Lab Anim (1987) 21:149–54. doi: 10.1177/002367728702100212 2955169

[70] Moody LV Miyamoto Y Ang J Richter PJ Eckmann L. Evaluation of peroxides and chlorine oxides as disinfectants for chemical sterilization of gnotobiotic rodent isolators. J Am Assoc Lab Anim Sci (2019) 58:558–68. doi: 10.30802/AALAS-JAALAS-18-000130 PMC6774453 31319899

[71] Trec S De Virgilio M Christou C. An alternative to cylinder drums for entering supplies and maintaining germ-free rodent isolators. STAR Protoc (2022) 3:101511. doi: 10.1016/j.xpro.2022.101511 35776636 PMC9254594

[72] Laranjeira PR de Souza RQ Bronzatti JAG Graziano KU. Steam sterilization chemical indicators are not adequate for monitoring real steam sterilization cycles. PDA J Pharm Sci Technol (2020) 74:435–8. doi: 10.5731/pdajpst.2019.09886 32737242

[73] Kelkar U Bal AM Kulkarni S. Monitoring of steam sterilization process by biologic indicators–a necessary surveillance tool. Am J Infect Control. (2004) 32:512–3. doi: 10.1016/j.ajic.2004.07.005 15609450

[74] Taconic Biosciences. Ensuring Sterile Feed for Germ-Free Mice. Available at: https://www.taconic.com/taconic-insights/microbiome-and-germ-free/sterile-feed-for-germ-free-mice.html (Accessed 30 May 2023).

[75] National Research Council (US), Subcommittee on Laboratory Animal Nutrition. Nutrient Requirements of Laboratory Animals. 4th ed. Washington (DC: National Academies Press (1995). 25121259

[76] Zucoloto AZ Yu IL McCoy KD McDonald B. Generation, maintenance, and monitoring of gnotobiotic mice. STAR Protoc (2021) 2(2):100536. doi: 10.1016/j.xpro.2021.100536 34027493 PMC8132126

[77] Qv L Yang Z Yao M Mao S Li Y Zhang J. Methods for establishment and maintenance of germ-free rat models. Front Microbiol (2020) 11:1148. doi: 10.3389/fmicb.2020.01148 32670216 PMC7326071

[78] Tusnio A Taciak M Barszcz M Paradziej-Łukowicz J Olędzka I Wiczkowski W. Thermal sterilization affects the content of selected compounds in diets for laboratory animals. J Anim Feed Sci (2014) 23(4):351–60. doi: 10.22358/jafs/65672/2014

[79] Prasain JK Wilson LS Arabshahi A Grubbs C Barnes S. Mass spectrometric evidence for the modification of small molecules in a cobalt-60-irradiated rodent diet. J Mass Spectrom (2017) 52(10):707. doi: 10.1002/jms.3981 29076237

[80] Daly MJ Minton KW. Recombination between a resident plasmid and the chromosome following irradiation of the radioresistant bacterium Deinococcus radiodurans. Gene (1997) 187:225–9. doi: 10.1016/s0378-1119(96)00755-x 9099885

[81] Sedlacek RS Rose EF. Steam quality and effective sterilization. Prog Clin Biol Res (1985) 181:65–8. 2862643

[82] Opfell JB. A general review of chemical sterilization in space research. Life Sci Space Res (1964) 2:385–405. 11883446

[83] Roth S Feichtinger J Hertel C. Characterization of Bacillus subtilis spore inactivation in low-pressure, low-temperature gas plasma sterilization processes. J Appl Microbiol (2010) 108:521–31. doi: 10.1111/j.1365-2672.2009.04453.x 19659696

[84] Zhang Z Jiang B Liao X Yi J Hu X Zhang Y. Inactivation of Bacillus subtilis spores by combining high-pressure thermal sterilization and ethanol. Int J Food Microbiol (2012) 160:99–104. doi: 10.1016/j.ijfoodmicro.2012.10.009 23177048

[85] Steoud RH Lyle RG. Contamination Control By Use of Ethylene Oxide. Washington DC Washington, D.C. Exotech Systems, Inc: Technology Summary (1972). Available at: https://ntrs.nasa.gov/api/citations/19720013398/downloads/19720013398.pdf.

[86] Camilleri E Korza G Green J Yuan J Li Y-Q Caimano MJ. Properties of aged spores of Bacillus subtilis. J Bacteriol (2019) 201:e00231–19. doi: 10.1128/JB.00231-19 PMC6597383 31061168

[87] Ganaway JR Allen AM Moore TD Bohner HJ. Natural infection of germfree rats with mycoplasma pulmonis. J Infect Dis (1973) 127:529–37. doi: 10.1093/infdis/127.5.529 4735425

[88] Fontaine CA Skorupski AM Vowles CJ Anderson NE Poe SA Eaton KA. How free of germs is germ-free? Detection of bacterial contamination in a germ free mouse unit. Gut Microbes (2015) 6:225–33. doi: 10.1080/19490976.2015.1054596 PMC4615677 26018301

[89] Pollard M Matsuzawa T. Radiation-induced leukemia in germfree mice. Proc Soc Exp Biol Med (1964) 116:967–71. doi: 10.3181/00379727-116-29423 14230401

[90] Kajima M Pollard M. Detection of viruslike particles in germ-free mice. J Bacteriol (1965) 90:1448–54. doi: 10.1128/jb.90.5.1448-1454.1965 PMC315834 4284971

[91] Pollard M Sharon N Teah BA. Congenital lymphocytic choriomeningitis virus infection in gnotobiotic mice. Proc Soc Exp Biol Med (1968) 127:755–61. doi: 10.3181/00379727-127-32793 5651129

[92] Pollard M. Spontaneous “Secondary” Disease in germfree AKR mice. Nature (1969) 222:92–4. doi: 10.1038/222092b0 5775840

[93] Megli CJ Coyne CB. Infections at the maternal-fetal interface: an overview of pathogenesis and defence. Nat Rev Microbiol (2022) 20:67–82. doi: 10.1038/s41579-021-00610-y 34433930 PMC8386341

[94] Theriault B Wang Y Chen L Vest A Bartma C Alegre M-L. Long-term maintenance of sterility following skin transplantation in germ-free mice. Transplant Direc (2015) 1:e28. doi: 10.1097/TXD.0000000000000539 PMC4655119 26609546

[95] Sekirov I Russell SL Antunes LCM Finlay BB. Gut microbiota in health and disease. Physiol Rev (2010) 90:859–904. doi: 10.1152/physrev.00045.2009 20664075

[96] Browne HP Forster SC Anonye BO Kumar N Neville BA Stares MD. Culturing of “unculturable” Human microbiota reveals novel taxa and extensive sporulation. Nature (2016) 533:543–6. doi: 10.1038/nature17645 PMC4890681 27144353

[97] Auchtung JM Robinson CD Britton RA. Cultivation of stable, reproducible microbial communities from different fecal donors using minibioreactor arrays (MBRAs). Microbiome (2015) 3:42. doi: 10.1186/s40168-015-0106-5 26419531 PMC4588258

[98] Russotto V Cortegiani A Raineri SM Giarratano A. Bacterial contamination of inanimate surfaces and equipment in the intensive care unit. J Intensive Care (2015) 3:54. doi: 10.1186/s40560-015-0120-5 26693023 PMC4676153

[99] Myemba DT Bwire GM Sangeda RZ. Microbiological quality of selected local and imported non-sterile pharmaceutical products in Dar Es Salaam, Tanzania. Infect Drug Resist (2022) 15:2021–34. doi: 10.2147/IDR.S355331 PMC9038149 35480052

[100] Bhatta DR Hamal D Shrestha R Hosuru Subramanya S Baral N Singh RK. Bacterial contamination of frequently touched objects in a tertiary care hospital of Pokhara, Nepal: how safe are our hands? Antimicrob Resist Infect Control (2018) 7:97. doi: 10.1186/s13756-018-0385-2 30128144 PMC6091187

[101] Matinyi S Enoch M Akia D Byaruhanga V Masereka E Ekeu I. Contamination of microbial pathogens and their antimicrobial pattern in operating theatres of Peri-Urban Eastern Uganda: A cross-sectional study. BMC Infect Dis (2018) 18:460. doi: 10.1186/s12879-018-3374-4 30200891 PMC6131813

[102] Sobel J Tucker N Sulka A McLaughlin J Maslanka S. Foodborne botulism in the United States, 1990-2000. Emerg Infect Dis (2004) 10:1606–11. doi: 10.3201/eid1009.030745 PMC3320287 15498163

[103] Hyun HH Zeikus JG Longin R Millet J Ryter A. Ultrastructure and extreme heat resistance of spores from thermophilic clostridium species. J Bacteriol (1983) 156:1332–7. doi: 10.1128/jb.156.3.1332-1337.1983 PMC217984 6643392

[104] Setlow P. Spore resistance properties. Microbiol Spectr (2014) 2(5). doi: 10.1128/microbiolspec.TBS-0003-2012 26104355

[105] Weber DJ Rutala WA Miller MB Huslage K Sickbert-Bennett E. Role of hospital surfaces in the transmission of emerging health care-associated pathogens: norovirus, clostridium difficile, and acinetobacter species. Am J Infect Control. (2010) 38:S25–33. doi: 10.1016/j.ajic.2010.04.196 20569853

[106] Kieninger M Mandlinger A Doblinger N Kieninger B Bele S Salzberger B. Impact of the implementation of a standard for preanalytical handling of samples for microbiological diagnostics on the quality of results at a neurocritical care unit. Med (Baltimore) (2021) 100:e27060. doi: 10.1097/MD.0000000000027060 PMC10545238 34449497

[107] ICR-Swab. Available at: https://www.merckmillipore.com/DE/de/products/industrial-microbiology/environmental-monitoring/environmental-monitoring-for-pharmaceutical-and-cosmetics-industry/surface-and-personnel-monitoring/icr-swabs/ScGb.qB.fycAAAFAXu1kiQpx,nav?ReferrerURL=https%3A%2F%2Fwww.google.com%2F (Accessed 31 May 2023).

[108] Sandle T. A review of cleanroom microflora: types, trends, and patterns. PDA J Pharm Sci Technol (2011) 65:392–403. doi: 10.5731/pdajpst.2011.00765 22293526

[109] Choo JM Leong LEX Rogers GB. Sample storage conditions significantly influence faecal microbiome profiles. Sci Rep (2015) 5:16350. doi: 10.1038/srep16350 26572876 PMC4648095

[110] Roesch LFW Casella G Simell O Krischer J Wasserfall CH Schatz D. Influence of fecal sample storage on bacterial community diversity. Open Microbiol J (2009) 3:40–6. doi: 10.2174/1874285800903010040 PMC2681173 19440250

[111] Shaw AG Sim K Powell E Cornwell E Cramer T McClure ZE. Latitude in sample handling and storage for infant faecal microbiota studies: the elephant in the room? Microbiome (2016) 4:40. doi: 10.1186/s40168-016-0186-x 27473284 PMC4967342

[112] Coico R. Gram staining. Curr Protoc Microbiol (2005) (1):A.3C.1–A.3C.2. doi: 10.1002/9780471729259.mca03cs00. Appendix 3, Appendix 3C. 18770544

[113] Luckey T ed. Germfree Life and Gnotobiology. 1st ed. Amsterdam: Elsevier Sciece (2012).

[114] Odds FC. Sabouraud(‘s) Agar. J Med Vet Mycol (1991) 29:355–9. doi: 10.1080/02681219180000581 1815027

[115] Dowell VR ed. Laboratory Methods in Anaerobic Bacteriology. Atlanta, Georgia: U.S. Department of Health, Education, and Welfare, Public Health Service, Center for Disease Control (1974).

[116] Dulbecco R Freeman G. Plaque production by the polyoma virus. Virology (1959) 8:396–7. doi: 10.1016/0042-6822(59)90043-1 13669362

[117] Eagle H. Amino acid metabolism in mammalian cell cultures. Science (1959) 130:432–7. doi: 10.1126/science.130.3373.432 13675766

[118] Rosenow EC. Elective localization and focal infection from oral sepsis. Dent Regist (1919) 73:557–67. PMC7845802 33703338

[119] Mangels JI Douglas BP. Comparison of four commercial brucella agar media for growth of anaerobic organisms. J Clin Microbiol (1989) 27:2268–71. doi: 10.1128/jcm.27.10.2268-2271.1989 PMC267008 2584378

[120] Brewer JH. Clear liquid mediums for the “Aerobic” cultivation of anaerobes. J Am Med Assoc (1940) 115:598–600. doi: 10.1001/jama.1940.72810340001009

[121] Bertani G. Studies on lysogenesis. I. The mode of phage liberation by lysogenic Escherichia coli. J Bacteriol (1951) 62:293–300. doi: 10.1128/jb.62.3.293-300.1951 14888646 PMC386127

[122] Macconkey A. Lactose-fermenting bacteria in faeces. J Hyg (Lond) (1905) 5:333–79. doi: 10.1017/s002217240000259x PMC2236133 20474229

[123] Macconkey AT. Bile salt media and their advantages in some bacteriological examinations. J Hyg (Lond) (1908) 8:322–34. doi: 10.1017/s0022172400003375 PMC2167122 20474363

[124] Ebeling AH. The permanent life of connective tissue outside of the organism. J Exp Med (1913) 17:273–85. doi: 10.1084/jem.17.3.273 PMC2125041 19867643

[125] Carrel A. On the permanent life of tissues outside of the organism. J Exp Med (1912) 15:516–28. doi: 10.1084/jem.15.5.516 PMC2124948 19867545

[126] Hirsch A Grinsted E. Methods for the growth and enumeration of anaerobic spore-formers from cheese, with observations on the effect of nisin. J Diary Res (1954) 21:101–10. doi: 10.1017/S0022029900007196

[127] MacFaddin JF ed. Media for Isolation - Cultivation - Identification - Maintenance of Medical Bacteria. 1st ed Vol. Volume I.XI. Baltimore, London: Williams and Wilkins (1985).

[128] Tuttle AR Trahan ND Son MS. Growth and maintenance of Escherichia coli laboratory strains. Curr Protoc (2021) 1:e20. doi: 10.1002/cpz1.20 33484484 PMC8006063

[129] Janssen PH Yates PS Grinton BE Taylor PM Sait M. Improved culturability of soil bacteria and isolation in pure culture of novel members of the divisions acidobacteria, actinobacteria, proteobacteria, and verrucomicrobia. Appl Environ Microbiol (2002) 68:2391–6. doi: 10.1128/AEM.68.5.2391-2396.2002 PMC127570 11976113

[130] Davis KER Joseph SJ Janssen PH. Effects of growth medium, inoculum size, and incubation time on culturability and isolation of soil bacteria. Appl Environ Microbiol (2005) 71:826–34. doi: 10.1128/AEM.71.2.826-834.2005 PMC546801 15691937

[131] Bosshard PP. Incubation of fungal cultures: how long is long enough? Mycoses (2011) 54:e539–545. doi: 10.1111/j.1439-0507.2010.01977.x 21605185

[132] Faith JJ Rey FE O’Donnell D Karlsson M McNulty NP Kallstrom G. Creating and characterizing communities of human gut microbes in gnotobiotic mice. ISME J (2010) 4:1094–8. doi: 10.1038/ismej.2010.110 PMC2927777 20664551

[133] Packey CD Shanahan MT Manick S Bower MA Ellermann M Tonkonogy SL. Molecular detection of bacterial contamination in gnotobiotic rodent units. Gut Microbes (2013) 4:361–70. doi: 10.4161/gmic.25824 PMC3839980 23887190

[134] Smith CJ Osborn AM. Advantages and limitations of quantitative PCR (Q-PCR)-based approaches in microbial ecology. FEMS Microbiol Ecol (2009) 67:6–20. doi: 10.1111/j.1574-6941.2008.00629.x 19120456

[135] Brielmeier M Mahabir E Needham JR Lengger C Wilhelm P Schmidt J. Microbiological monitoring of laboratory mice and biocontainment in individually ventilated cages: a field study. Lab Anim (2006) 40(3):247–60. doi: 10.1258/002367706777611497 16803642

[136] Jacoby RO Smith AL. Mouse parvovirus: survival of the fittest. Comp Med (2003) 53(5):470–1. 14655987

[137] Joh J Proctor ML Ditslear JL King WW Sundberg JP Jenson AB. Epidemiological and phylogenetic analysis of institutional mouse parvoviruses. Exp Mol Pathol (2013) 95(1):32–7. doi: 10.1016/j.yexmp.2013.03.009 23545399

[138] Karst SM Wobus CE Lay M Davidson J Virgin HW 4th. STAT1-dependent innate immunity to a Norwalk-like virus. Science (2003) 299(5612):1575–8. doi: 10.1126/science.1077905 12624267

[139] Manuel CA Hsu CC Riley LK Livingston RS. Soiled-bedding sentinel detection of murine norovirus 4. J Am Assoc Lab Anim Sci (2008) 47(3):31–6. PMC2654006 18459710

[140] Natural pathogens of laboratory mice, rats, and rabbits and their effects on research. Clin Microbiol Rev (1998) 11(2):231–66. doi: 10.1128/CMR.11.2.231 PMC106832 9564563

[141] Magan N Evans P. Volatiles as an indicator of fungal activity and differentiation between species, and the potential use of electronic nose technology for early detection of grain spoilage. J Stored Prod Res (2000) 36(4):319–40. doi: 10.1016/s0022-474x(99)00057-0 10880811

[142] Suchorab Z Frąc M Guz Ł Oszust K Łagód G Gryta A. A method for early detection and identification of fungal contamination of building materials using e-nose. PloS One (2019) 14(4):e0215179. doi: 10.1371/journal.pone.0215179 30964926 PMC6456197

[143] Siller-Ruiz M Hernández-Egido S Sánchez-Juanes F González-Buitrago JM Muñoz-Bellido JL. Fast methods of fungal and bacterial identification. MALDI-TOF mass spectrometry, chromogenic media. Métodos rápidos de identificación de bacterias y hongos. Espectrometría de masas MALDI-TOF, medios cromogénicos. Enferm Infecc Microbiol Clin (2017) 35(5):303–13. doi: 10.1016/j.eimc.2016.12.010 28108122

